# Radiotherapy of Follicular Lymphoma: Updated Role and New Rules

**DOI:** 10.1007/s11864-014-0286-4

**Published:** 2014-04-04

**Authors:** Joachim Yahalom

**Affiliations:** Department of Radiation Oncology, Memorial Sloan-Kettering Cancer Center, 1275 York Avenue, New York, NY 10065 USA

**Keywords:** Non-Hodgkin lymphoma, Low-grade lymphoma, Follicular lymphoma, Nodal marginal zone lymphoma, Marginal zone lymphoma, Radiotherapy, Involved-site radiotherapy, Involved-field radiotherapy, Low-dose radiation

## Abstract

Radiation is established as one of the most powerful, highly effective treatments for non-Hodgkin lymphoma (NHL). Unfortunately, in recent years the medical oncology community has improperly underutilized radiotherapy (RT) in the management of NHL. Replacing RT with longer chemotherapy and/or immunotherapy may not necessarily be a good alternative approach and may lead to suboptimal outcome and more toxicity, particularly in patients with localized disease. Some misconceptions regarding the use of RT emanated from the ways RT has been utilized in the past—as a single therapy and in high doses and large fields. Major developments in imaging technology, radiation planning concepts, and RT precision and delivery have been revolutionized RT for NHL over the past two decades. Modern proper administration should result with very minimal acute or late side effects. Some of the controversial issues of the use of RT borrowed from Hodgkin lymphoma, such as risk of secondary tumors, are irrelevant to patients with NHL but cause unnecessary patient and physician scare. Many lymphoma types are notoriously sensitive to RT, especially the indolent types. When localized, like in most marginal zone lymphoma (MZL) and almost a third of follicular lymphomas (FL), RT is potentially curative, even with low dose and small volumes. In more aggressive lymphomas, RT often is an effective consolidation after chemotherapy in complete or even incomplete responders. It also is an important component of salvage and palliation. In older patients, RT is particularly valuable, because chemotherapy tolerance and salvage options may be limited. The International Lymphoma Radiation Oncology Group (ILROG) developed and published modern guidelines for using RT in NHL, including FL. The guidelines emphasize the new concept of RT field: involved site radiotherapy (ISRT). These modern ILROG principles and several relevant studies that looked into the proper integration of RT in the management of NHL patients are the focus of this manuscript.

## Introduction

Ionizing radiation is a highly effective modality for the treatment of most lymphomas. The dramatic effects of radiation alone in reducing large lesions and even eliminating Hodgkin lymphoma (HL) lesions were reported more than 112 years ago, soon after the discovery of x-rays by Wilhelm Conrad Roentgen. Yet, during the first half of the 20th century, when all lymphomas remained incurable, radiation was used mostly for palliation and responses where brief due to technical constraints and/or poor methods of delivering radiation. As x-ray technology improved in the 1940s and the concept of irradiating beyond the involved area was adopted, patients with early-stage HL and non-Hodgkin lymphoma (NHL) could be cured with radiation alone—the only effective curative modality for lymphomas that was available until the late 1960s.

Before the advent of effective chemotherapy, attempts were made to cure NHL by maximizing the use of radiation alone. Optimizing the selection of patients and tailoring the radiation fields was associated with aggressive staging efforts that included using staging laparotomy for HL and even NHL. Moreover, the dependency of RT as a single modality required wide extension of the radiation field as well as raising the dose to normal tissue tolerance levels. While this radical radiotherapy led to the cure of many patients, it also was associated with late development of complications and increased the mortality of cured patients beyond what is expected of the normal population. This was the price of successfully pioneering radiation therapy as a curative modality before the availability of effective chemotherapy.

The emergence of more effective and less toxic chemotherapy over the past three decades led to considerable changes in the use of RT in the management of lymphomas. First, in several types of NHL, chemotherapy has become the primary modality with radiotherapy used for consolidation and reduction of relapse risk. Yet, in some lymphomas, mostly early-stage, low-grade lymphomas where chemotherapy is less effective, radiation alone remained the standard of care. Radiation alone is currently the treatment of choice for localized follicular lymphoma (FL) and marginal zone lymphoma (MZL) lymphomas.

Localized stages of FL (I and contiguous II) occur in approximately 22 % to 33 % of all cases [1, 2]. Some of these early-stage patients may have a normal bone marrow examination by morphology, but sensitive essays may detect a clonal population of B cells in the blood and/or bone marrow. The clinical significance of this finding in patients treated with local therapy is still uncertain.

Early-stage patients present often with a median age of 60 years, usually with a good performance status, no systemic symptoms, and normal LDH. The disease is likely to be confined to one anatomic region, often the neck or inguinal area. Involvement of an extranodal site is less common, only 25 % of patients [3].

Finally, radiotherapy is an excellent palliative modality that provides long-term local control and clinical benefit even for lymphomas in advanced-stage, such as follicular lymphoma, small lymphocytic lymphoma, MZL, and mantle cell lymphoma. These specific types of lymphoma are highly sensitive to radiation and very low doses may be adequate for extended local control [4].

## The rationale for using RT in follicular lymphoma

For patients with stage I or contiguous stage II follicular lymphoma RT alone is well recognized, “preferred,” or the treatment of choice. [5] Several centers reviewed the long-term outcome of RT alone and reported relapse-free survival in the range of 50 % at 10 years and approximately 40 % at 15 years; overall survival rates were 60-80 % and 35-45 %, respectively. Median survival in some series was 19 years. Thus, RT is likely curative for a significant number of patients with early-stage FL [2, 3].

The largest retrospective study of 6,568 patients with follicular lymphoma stage I or II diagnosed between 1973 and 2004 was based on SEER data [6]. In 34 % of these patients, RT was used as the initial treatment. The group receiving RT at the onset had higher rates of disease-specific survival at 5 years (90 vs. 81 %), 10 years (79 vs. 66 %), 15 years (68 vs. 57 %), and 20 (63 vs. 51 %) years. Overall survival also was improved for patients who received initial radiation therapy. With RT, most of the lesions completely regress and local relapse at an irradiated site is rare. Relapses usually occur distant from the RT site and are rare after 10 years (1-11 %) [7, 8]. Risk factors for relapse are age (>60 years), abnormal LDH, and bulk.

These data support the notion that a substantial subset of patients with limited-stage follicular lymphoma may be cured with radiotherapy alone. Despite evidence supporting the use of radiation, the majority of patients with stage I disease treated in the United States do not receive radiation therapy [9]. The National LymphoCare Study (funded in part by Genentech and Biogen) assembled patients with early-stage FL patients from several institutions. The authors reported that despite NCCN guidelines recommending RT alone as the preferred approach, only 23 % of the patients received RT as their initial treatment [5]. Yet, an additional 20 % received RT within 90 days after chemotherapy and/or rituximab either because of inadequate response/progression or possibly as part as planned combined modality therapy. Surprisingly, 30 % of patients were “observed” possibly based on very limited data of highly selected 43 patients from Stanford who were not treated and had comparable outcome to the majority who received RT. [10] Potentially misleading, in my opinion, is the second report from the LymphoCare group of 206 patients that were rigorously staged to have only one site of disease and of whom selectively only 27 % received RT, 17 % were observed, 13 % had RT and another systemic treatment, 28 % had rituximab and chemotherapy, and 12 % had rituximab alone [11]. At a median follow-up of 5 years, no differences in progression-free survival (PFS) between the different treatments selected for these patients. The retrospective nature and almost certain selection bias for treatment should be highlighted before making any conclusions on treatment choices from this small study. Interestingly, the study also compared patients that were rigorously staged to those that were considered stage I without appropriate staging. The rigorously staged patients had significantly better PFS compared with those that have not been fully staged, even after adjustment to patient, disease, and treatment factors.

Clearly, the definition of stage I have changed over time and stage migration phenomena are relevant when discussing results of RT alone from older series. The original series probably included of unknown fraction of patients with more advanced stages and thus the relapse rate with localized therapy is expected to be superior for current properly staged patients. One caveat, however, is that older RT fields were significantly larger (and thus more toxic) than today but could have controlled more nodal sites that could potentially still relapse with today’s minimal RT.

Another issue that has not been addressed as yet in early-stage FL is the effect of treatment on the risk of transformation into aggressive lymphoma. It is logical to expect that patients whose localized disease has been decimated by RT are less likely to transform. This question has not been addressed yet by any study. Yet, it remains of concern that in the Stanford retrospective follow-up of 43 patients with stage I-II FL that were selected to no initial treatment, 4 patients have transformed into higher-grade lymphoma [10].

## Lower RT dose for indolent lymphomas

The indolent lymphomas (FL, MZL, CLL/SLL) as well as mantle cell lymphoma (MCL) are inquisitively radiosensitive. Yet, in the past, many clinicians used radiation doses in the range of 36-45 Gy to treat all types of lymphoma based on some very old, questionable, dose-response studies [12]. More recently, an important randomized study from the United Kingdom compared the former standard dose of 40-45 Gy to 24 Gy in 361 involved sites of patients with indolent lymphomas (mostly FL and MZL in early stages) [13•]. At a median follow-up of 5.6 years, there was no difference in overall response (93 %/92 %) between the standard and lower dose arms. There was no difference in the rate of within radiation field progression, progression-free survival, or overall survival. Based on this seminal study, the RT dose recommended for most cases of localized FL is 24 Gy [5, 14•]. Exceptions that may suggest the employment a higher dose (30-36 Gy) are bulky disease >5 cm and grade IIIA pathology. For the relatively rare nodal MZL behave clinically and are managed according to stage like FL, we also recommend ISRT of 24-30 Gy. The more common localized extranodal MZL (MALT lymphomas) also are very sensitive to RT and should be managed in the same low range of 24-30 Gy (see NCCN Guidelines [5]).

During the past 20 years, very low doses or RT of only 2 Gy X2 (known in RT jargon as “*boom-boom”*) were shown to be highly effective when used for palliation of advanced-stage, relapsing, or even post multiple chemotherapy refractory patients with indolent lymphomas and MCL [15]. Amazingly, in many cases this innocent RT dose resulted in complete local lasting responses.

The rewarding *“boom-boom”* experience with a total dose of only 4 Gy led to a second UK randomized study called FORT. [16•] It was designed as noninferiority study and the primary question for analysis was time to local progression. A total of 614 sites in 548 patients with FL (and some with MZL) were prospectively randomized to receive RT of either the newly established dose of 24 Gy or the very low dose of 4 Gy. In 60 % the intent of RT was considered as palliative and in 40 % as curative.

The results have been reported recently and clearly showed that 4 Gy was inferior to 24 Gy in terms of time to local progression (hazard ratio of 3.4; 95 confidence interval [CI] 2.09-5.55, *p* < 0.0001). Median follow-up was 26 months, with a range that extended to 75 months. The failure to demonstrate noninferiority of the lower dose is particularly important in the curative setting of previously untreated localized disease. The difference for the curative intent subgroup also was significantly better for the higher dose. Thus, 24 Gy remains the standard dose for curative treatment of localized disease until a lower dose (that is in between than 4 Gy and 24 Gy) is explored properly. In this large study, grade 3 acute or late side effects were rare (4 Gy: acute 1.3 %, late 1.3 %; 24 Gy: acute 2.8 %, late 1.4 %). There was only grade 4 event of myelosuppression.

Although the dose of 24 Gy also yielded better results for local control duration in the palliative setting, the results with 4 Gy were quite impressive with almost 50 % of patients achieving a complete response and another 32 % having regression of more that 30 % of their disease [17•]. Thus, 4 Gy remains an excellent option for many patients in the palliative and relapse/setting. It often, in my opinion, serves the patient better that another round of systemic treatment or participation in phase I studies and should be practiced more frequently. Obviously “boom-boom” may be repeated multiple times; multiple sites or one large field may be treated at the same time and all that is required are only two visits to the RT facility.

## Modern RT (and smaller) field design for patients with follicular lymphoma: The involved site radiotherapy (ISRT) approach

The early RT studies used very large fields that often included irradiation of all involved and uninvolved lymph nodes and even the spleen; the field was called total lymphoid irradiation (TLI). This approach was developed at Stanford at the 1960s and resembled the approach to RT of Hodgkin lymphoma at the time. For the last three decades, it has been replaced by more limited fields of radiation that restricted the treatment to the lymph node groups on only one side of the diaphragm, such as the mantle field for involvement of the upper body lymph nodes and Inverted Y large field for the lower part. Although radiation that include fields on both side of the diaphragms resulted in a better freedom of relapse in the era of primitive staging, there was no difference in overall survival and salvage options were limited by irradiation to full dose to a large part of the bone marrow space [18].

Radiation fields later included only the regional or the involved fields; adjacent lymph node areas to the involved node/nodes were irradiated and field borders were determined by anatomical landmarks, often skeletal structure that could easily be identified by fluoroscopy technique used to plan the treatment.

With the advent of CT simulation often with data from FDG-PET-CT, localization of involved or suspicious lymph nodes was more precise and thus easier to target. Safer reduction of the RT volume and avoidance of normal structures as well more sophisticated treatment planning programs, such as three-dimensional conformal radiotherapy (3DCRT) and intensity-modulated intensity radiotherapy (IMRT) have become available in most centers.

A study from Vancouver looked retrospectively at patients that with limited-stage follicular lymphoma treated either to the involved region or to a smaller field limited to the involved site. [8] At a median follow-up of 7.5 years, both the larger and the smaller fields yielded excellent local control. Only 1 % of patients who were treated with the smaller “involved node” approach relapsed in adjacent regional nodes and there was no difference with respect to distant failure between the two groups (involved site-32 %, Regional RT-38 %).

To guide and harmonize field concepts and design, the International Lymphoma Radiation Oncology developed and recently published RT guidelines for nodal based NHL, including follicular lymphomas. [14•] These guidelines for volume and dose have been adopted by the NCCN and provide the basis for the current approach for using RT in lymphoma patients [5].

The recommended field contains only the involved site, is based on imaging information, and is influenced by clinical consideration, such as the availability of high-quality imaging before intervention that could have affected the information, such as excision or systemic treatment, on the imaging at the time of simulation, the histology and potential presence of microscopic disease, particularly if RT is the only modality that is used for curative purposes, as often is the case in localized FL.

The radiation oncologist is expected to exercises clinical judgment based on these principles to determine the CTV (clinical target volume) and employs ICRU principles to determine the PTV (planning treatment volume).

The end result of using the ISRT concept often is a RT field that is significantly smaller than the regional and involved-field practiced in the past. Yet, it is more generous than the very strict involved-node RT (INRT) that was designed for Hodgkin lymphoma patients that have optimal imaging data prior to any intervention that can be integrated into the postchemotherapy planning CT [19].

## Summary

New information based on randomized studies allowed a significant reduction in RT dose to 24 Gy for potentially curative cases. Increasing experience that supports the benefit from very small dose of 4 Gy for palliative or heavily pretreated, relapsed, or refractory patients. The lower dose approach made radiation safer and easier to administer to many patients in various stages of FL. Most side effects concerns of the past, such as dryness of the mouth or other organ malfunction, are irrelevant with this low-dose small volume approach. Limiting and smartly determining the RT field according to ILROG guidelines allows using RT with a better therapeutic ratio than in the past. The very large studies that documented that RT in NHL patients do not have an increased risk of second malignancies also are reassuring. Radiation should remain an important modality for the best care of FL patients. Oncologists should make the best use of its availability, particularly in patients with indolent lymphomas.
